# Case Report: Safety and efficacy of combination therapy with adalimumab and anakinra in two patients with severe inflammatory diseases

**DOI:** 10.3389/fped.2026.1713890

**Published:** 2026-04-15

**Authors:** Simone Coslovich, Matteo Bramuzzo, Serena Pastore, Daniela Chicco, Sara Lega, Giulia Gortani, Thomas Caiffa, Alberto Tommasini, Andrea Taddio

**Affiliations:** 1 Department of Medicine, Surgery, and Health Sciences, University of Trieste, Trieste, Italy; 2 Institute for Maternal and Child Health, IRCCS “Burlo Garofolo”, Trieste, Italy

**Keywords:** combination biological therapy, efficacy, IL-1 inhibitor, inflammatory diseases, safety, TNF-α inhibitor

## Abstract

**Background:**

Chronic inflammatory diseases in children can involve distinct immunologic pathways, occasionally necessitating dual biologic therapy when single-agent treatment is insufficient. Evidence on combined IL-1 and TNF-α inhibition remains limited.

**Cases:**

A 15-year-old girl with ulcerative colitis and recurrent pericarditis received adalimumab and anakinra, achieving clinical remission without infections over eight months. An 11-year-old boy with juvenile idiopathic arthritis, uveitis, and colchicine-resistant familial Mediterranean fever received combined adalimumab and anakinra, achieving low disease activity in both conditions at six months, without adverse events.

**Discussion:**

Dual blockade of TNF-α and IL-1 may be rational in selected patients due to overlapping and complementary roles of these cytokines in driving inflammation. Our cases illustrate that this combination can be effective and well tolerated with careful monitoring.

**Conclusion:**

In carefully selected pediatric patients, simultaneous TNF-α and IL-1 inhibition may provide clinical benefit. Further studies are required to assess long-term safety and efficacy.

## Introduction

Chronic inflammatory diseases may have very debilitating symptoms due to its systemic involvement. In recent years, biologic drugs are increasingly used to reduce disease activity blocking selective cytokine pathways. In rare cases multiple inflammatory conditions may be present requiring a dual biological therapy since the activation of different inflammatory pathways, with a major risk of immunosuppression. We report two cases of concomitant inflammatory conditions successfully treated with dual biologic therapy without short-term side effects.

## Case description

### Case 1

A 15-year-old girl was diagnosed with ulcerative colitis (UC) based on a 1-year long lasting history characterized by bloody diarrhea, elevation in fecal calprotectin and typical endoscopic and histological findings. Since the condition was not controlled by oral mesalazine and oral prednisone therapy, adalimumab (40 mg every 15 days) was subsequently started while tapering corticosteroids.

60 days later, while the UC was in clinical and biochemical remission (PUCAI score 0, Physician's Global Assessment: remission), the patient was hospitalized due to the sudden onset of chest pain associated with weight loss and elevated inflammatory markers. Echocardiography revealed pericardial effusion, further investigations ruled out possible underlying diseases such as systemic lupus erythematosus, tuberculosis, and malignancies. Suspecting pericarditis due to mesalazine, the medication was discontinued. New episodes of pericarditis, however, continued to occur even while the UC was still under treatment. NSAIDs (non-steroidal anti-inflammatory drugs) and colchicine were started concurrently, but glucocorticoids were the only drug that could control the pericarditis. Nevertheless, every time the medication was stopped, the pericarditis recurred. To achieve better disease control and minimize the risk of side effects resulting from chronic glucocorticoids, anakinra (IL-1 inhibitor) was started at a dosage of 100 mg per day subcutaneously. Anakinra was selected rather than canakinumab because its short half-life allows rapid dose adjustment and discontinuation if needed, which may be advantageous when initiating dual biologic therapy. Adalimumab was selected over other anti-TNF agents for its established efficacy in pediatric UC and for the convenience of subcutaneous, facilitating outpatient management.

The patient did not experience any further episodes of pericarditis during the following eight months of combination treatment with adalimumab and anakinra and did not experience any side effect nor severe or invasive infection. Disease activity indices, including PUCAI and Physician's Global Assessment, confirmed clinical remission throughout treatment.

### Case 2

An 11-year-old boy with a history of left knee arthritis, elevated blood inflammatory markers, and antinuclear antibody (ANA) positive was diagnosed with oligoarticular juvenile idiopathic arthritis (JIA) and uveitis. Whilst arthritis was well controlled by intra-articular glucocorticoids alone, a systemic therapy with methotrexate and adalimumab was needed to control uveitis. Later, while the patient was still on adalimumab, several chest pain crises with high inflammation indices appeared, eventually leading to the diagnosis of Familial Mediterranean Fever (FMF), which was then confirmed by the finding of a homozygous M694 V mutation. Due to colchicine resistance, monthly injections of canakinumab were soon started, after stopping adalimumab, but unfortunately uveitis relapsed after a couple of months. Adalimumab was then added, with remission of uveitis but not FMF. For this reason, anakinra (100 mg per day subcutaneously) was added to adalimumab, chosen for its efficacy in controlling pediatric uveitis and established safety profile, while anakinra provided flexible daily control of FMF flares compared with canakinumab.

At six-month follow-up disease activity indices, including JADAS, CHAQ, AIDAI, and Physician's Global Assessment, confirmed low disease activity in both conditions.

No infections or adverse events were observed at six-month follow-up. The combination treatment of anakinra and adalimumab was well-tolerated, with no documented side effects at follow-up.

**Figure 1:**
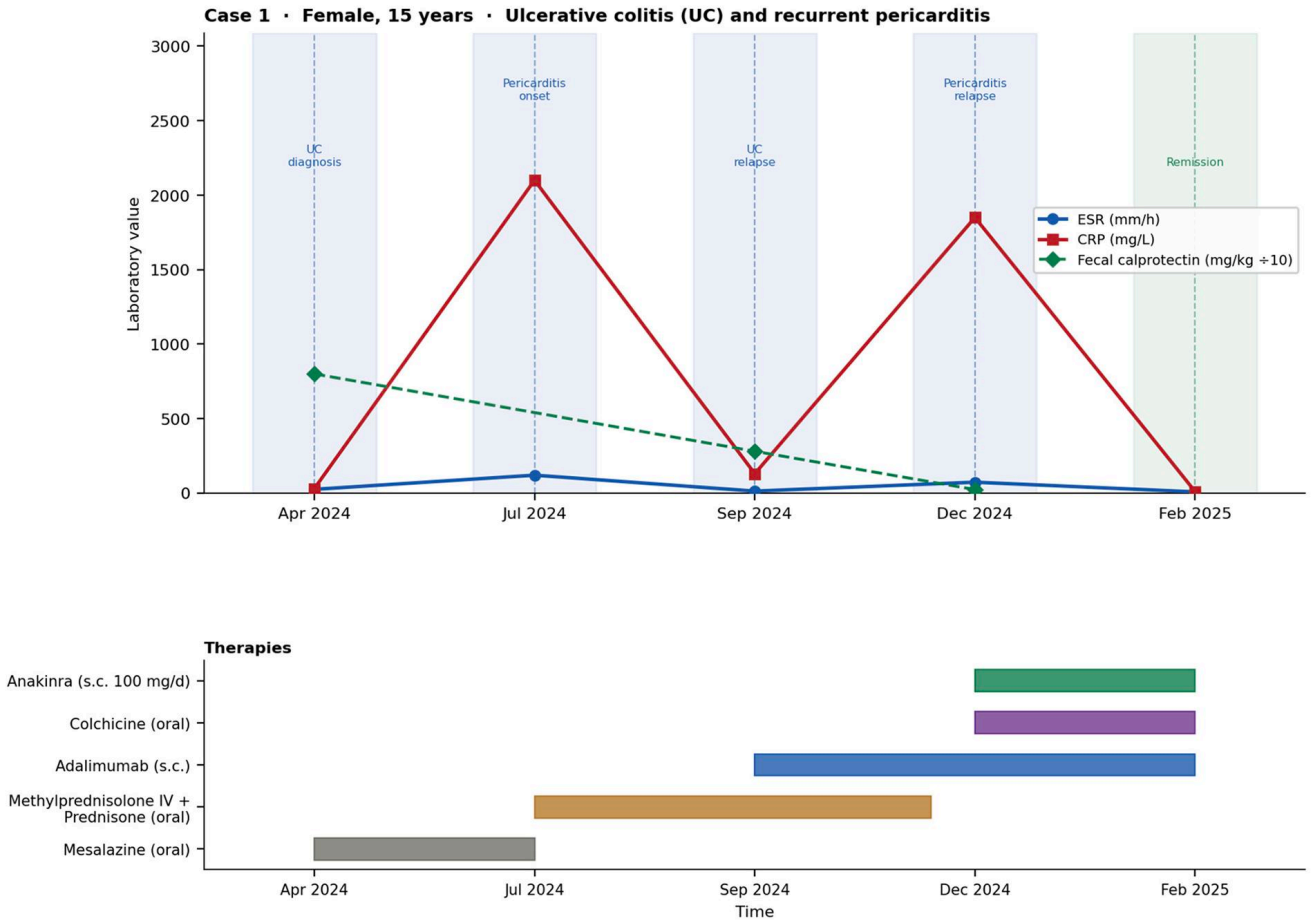
Clinical timeline of case 1 illustrating inflammatory marker trends and sequential therapeutic strategies leading to combined biologic therapy.

**Figure 2:**
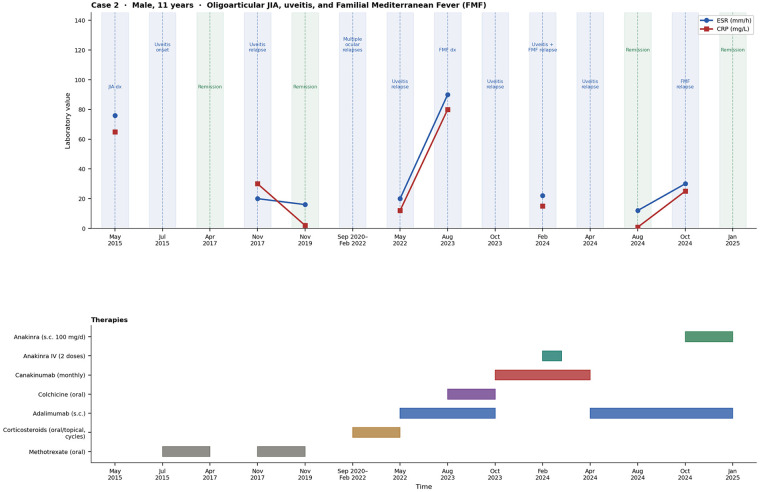
Clinical timeline of case 2 showing disease course, inflammatory markers (ESR and CRP), and major therapeutic interventions including initiation of Adalimumab and anakinra.

For both patients, baseline infectious screening was performed prior to the initiation of each biologic agent and before combination therapy, including Quantiferon-TB testing, CMV/EBV serology, hepatitis panel, and vaccination status assessment. Treatment response was assessed not only clinically but also through serial laboratory markers of systemic inflammation (CRP and ESR), disease-specific activity indices (PUCAI for UC; JADAS, CHAQ, and AIDAI for JIA/FMF), and organ-specific evaluations (echocardiography for pericarditis and ophthalmologic slit-lamp examination for uveitis). Improvement was defined as normalization or significant reduction of inflammatory markers together with achievement of clinical remission according to validated pediatric scores. The longitudinal clinical course of both patients, including key laboratory parameters (CRP, ESR and fecal calprotectin) and timing of biologic therapies, is summarized in Figures 1, 2.

Follow-up occurred every 4–6 weeks with structured clinical and laboratory monitoring, including CBC, CRP, ESR, and liver and kidney function tests. No routine antimicrobial prophylaxis was given, but any febrile or infectious symptoms were promptly evaluated.

## Discussion

Chronic inflammatory diseases may coexist in the same patient, worsening the overall clinical picture and making therapeutic management challenging. Biologic drugs are increasingly used to target selective cytokine pathways, and in rare situations distinct inflammatory mechanisms may require different agents simultaneously. IL-1 and TNF-α arise from distinct immunologic triggers, reflecting different facets of the inflammatory response. IL-1 is primarily generated through inflammasome activation, driven by the synergistic action of bacterial molecular patterns and damage-associated molecular patterns from cell injury. This mechanism is essential for rapidly kickstarting inflammation upon bacterial entry into the body. IL-1 can also stem from defective regulatory pathways, as observed in classic autoinflammatory diseases like familial Mediterranean fever (FMF) or recurrent pericarditis. In contrast, TNF-α production unfolds as part of a more complex and protracted inflammatory process that engages both innate and adaptive immunity. Dysregulation here manifests in conditions such as inflammatory bowel disease (IBD) and juvenile idiopathic arthritis (JIA). Despite these differences, the pathways frequently converge: TNF-α can upregulate inflammasome components, while IL-1β amplifies TNF-α-mediated transcriptional programs. This interplay likely contributes to the persistence of inflammation across tissues like serosal membranes, the intestine, and synovium (1–3). TNF-α and IL-1β participate in a bidirectional amplification loop: TNF-α enhances NF-*κ*B activation and promotes transcription of pro–IL-1β, while IL-1β further stimulates TNF-α production and sustains downstream inflammatory cascades. Although inhibition of one cytokine may partially dampen the other, this cross-regulation is often incomplete, particularly when distinct tissue compartments or immune triggers are involved. In such contexts, residual activity of the parallel pathway may maintain clinically relevant inflammation. This may be particularly relevant when different organs are driven by partially distinct immune mechanisms, as observed in our patients, in whom intestinal or ocular disease remained controlled while autoinflammatory manifestations persisted. Given this incomplete cross-regulation, persistent organ-specific inflammation in our patients despite guideline-based monotherapy at therapeutic doses and with adequate clinical response in the primary disease suggested the coexistence of partially independent pathogenic drivers rather than a single dominant cytokine axis. Circulating TNF-α and IL-1β levels were not measured in these patients. In routine clinical practice, cytokine quantification is not standardized, lacks clear correlation with tissue-level activity, and does not reliably guide therapeutic decisions. Therefore, treatment strategies were based on clinical phenotype, validated disease activity indices, and objective inflammatory markers rather than serum cytokine levels.In selected pediatric patients with simultaneous activation of these pathways, combined IL-1 and TNF-α blockade may help interrupt parallel inflammatory circuits when single-agent therapy is insufficient. The pediatric immune system differs from the adult counterpart in terms of innate–adaptive balance and cytokine responsiveness, reflecting the progressive maturation of immunoregulatory networks during early life (4). Children may therefore exhibit overlapping autoinflammatory and autoimmune features within the same clinical course, reflecting the continuum between innate and adaptive immune dysregulation described in several pediatric inflammatory disorders and potentially explaining heterogeneous or incomplete responses to single-cytokine blockade (5, 6). This immunologic plasticity may partly account for cases in which targeting a single pathway fails to fully control disease activity, as observed in our two patients where concomitant inflammatory conditions required dual therapeutic targeting. Evidence on dual biologic therapy – particularly the combination of IL-1 and TNF-α inhibitors – remains limited and sometimes conflicting, raising concerns about safety. Most safety data in pediatric populations derive from studies on biologic monotherapy, which generally show good tolerability but confirm infections as the most frequent adverse events requiring careful monitoring (7, 8). Combination therapy with etanercept (TNF-α inhibitor) and anakinra was evaluated for the treatment of rheumatoid arthritis in adults: it provided no added benefit and increased the incidence of infections for combination therapy (9). In a study of 4 children with refractory systemic JIA, treatment with anakinra plus abatacept was initiated, with clinical improvement and no occurrence of severe infections (10). Two of the four adult patients with dual biologic therapy for FMF and spondyloarthritis were treated with an IL-1 inhibitor (anakinra or canakinumab) and a TNF-α inhibitor (etanercept and certolizumab) without any adverse effects nor severe infection (11). A combination of two biologic drugs has also been proposed for the treatment of refractory inflammatory bowel disease, even in the absence of other inflammatory comorbidities. Evidence for dual biologic therapy is emerging in both adult and pediatric populations. Reported combinations have most commonly involved anti-TNF-α agents (such as adalimumab) with ustekinumab (IL-12/23 inhibitor), vedolizumab (*α*4*β*7 integrin antagonist), or JAK inhibitors (tofacitinib or upadacitinib), and have generally appeared safe and effective, although adverse events such as infections or elevated liver enzymes have occasionally led to treatment discontinuation (12–15).

We report our small experience with the combined use of adalimumab and anakinra in two patients with concomitant inflammatory diseases involving distinct pathogenic pathways (FMF and JIA with uveitis; UC and recurrent pericarditis). In both cases, dual biologic therapy resulted in improved disease control without evidence of increased infections or other short-term immunosuppressive complications. Treatment efficacy was supported not only by clinical assessment but also by objective findings, including normalization or significant reduction of inflammatory markers (CRP and ESR) alongside validated pediatric disease activity indices. This study has several limitations, including the small sample size, short follow-up, limited generalizability, lack of pharmacokinetic data, and its observational design. Although follow-up was limited to 6–8 months, dual therapy is currently planned to continue as long as sustained clinical remission is maintained and no safety concerns arise. In the case of prolonged stable remission, a cautious stepwise de-escalation strategy may be considered, guided by careful reassessment of the predominant inflammatory pathway. Conversely, treatment modification or discontinuation would be indicated in the event of serious infections, significant adverse events, loss of efficacy, or patient/family preference. Given the absence of standardized recommendations for dual biologic therapy in pediatric patients, treatment duration must be individualized and periodically re-evaluated. A structured long-term monitoring strategy is therefore essential and includes regular clinical assessments, laboratory monitoring (complete blood count, liver and kidney function tests, CRP, and ESR), vaccination status review, and prompt evaluation of any febrile or infectious symptoms to enable early detection of infections, malignancy, or other adverse events.

## Conclusion

The combined use of two biologic drugs remains debated because of persistent concerns regarding both safety and long-term efficacy. However, in our two patients – in whom adalimumab and anakinra were administered together to control severe and highly disabling inflammatory diseases – this dual cytokine blockade was well tolerated and free of evident side effects over the follow-up period. In selected scenarios, simultaneous inhibition of TNF-*α* and IL-1 may be rational due to overlapping and complementary roles of these cytokines in driving inflammation. In such cases, careful clinical monitoring and prompt recognition of any complications remain essential. Further studies are needed to confirm the safety and efficacy of dual biologic therapy in pediatric populations.

## Data Availability

The raw data supporting the conclusions of this article will be made available by the authors, without undue reservation.
